# 

**Published:** 1886-11-06

**Authors:** 


PRICE ONE PENNY.

No. 6, Vol. I.

November 6th, 1886.

Per Annum, 6s. 6d., post free.

Monthly Parts, 6d.; post free, 7½d.

Ditto per Ann., 7s. 6d., post free.

